# Nursing Doctorate Issues, Challenges and Expected Changes Across Europe: A Rapid Review and Experts' Opinion

**DOI:** 10.1111/jan.70300

**Published:** 2025-10-28

**Authors:** Alvisa Palese, Gülcan Taşkiran Eskici, Katerina Bitolkoska, Gaia Dussi, Gaia Magro, Chiara Moreal, Stefania Chiappinotto, Anna Pilewska‐Kozak, Beata Dobrowolska

**Affiliations:** ^1^ Department of Medicine University of Udine Udine Italy; ^2^ Department of Nursing Management Faculty of Health Sciences, Ondokuz Mayis University Samsun Turkey; ^3^ Azienda Sanitaria Friuli Occidentale Pordenone Italy; ^4^ Department of Obstetrics and Gynecology Nursing, Chair of Obstetrics and Gynaecology Faculty of Health Sciences, Medical University Lubin Poland; ^5^ Department of Holistic Care and Management in Nursing, Faculty of Health Sciences Medical University of Lublin Lubin Poland

**Keywords:** doctoral education, Europe, nurse education, online survey, rapid review

## Abstract

**Aims:**

Updating recent reviews and enriching the available evidence with expert opinions on the challenges and expected reforms needed in doctoral education across Europe.

**Design:**

A dual design based on a rapid review and an online survey.

**Data Source/Review Method:**

The PubMed, CINAHL and Scopus databases were searched for studies published between January 2020 and June 2025 using the terms “PhD” AND “nursing”. In parallel, an online survey with open‐ended questions was distributed to a purposive sample of academic experts in each European country. Findings from the literature were juxtaposed and integrated with the data from the expert survey and integrated.

**Results:**

A total of 23 studies and 26 expert opinions. Doctoral nursing education in Europe is facing seven key challenges regarding: (1) institutions and their structure, (2) supervision, (3) candidates, (4) research process and outcomes, (5) professional development and career progression, (6) international collaboration and (7) paradigm‐related concerns. Six anticipated changes/recommendations were identified in (1) structural and policy reforms, (2) supervision and mentoring, (3) candidate recruitment, retention and support, (4) financial and institutional support, (5) professional development and career recognition, (6) collaboration and internationalisation. While some challenges and changes were confirmed by the literature, others emerged from the experts' insights.

**Conclusions:**

Complex challenges are faced by European doctoral nursing education, some under‐researched as issues of supervision and candidate experience. Strengthening structures, mentorship and international collaboration is essential to align education with academic standards and healthcare needs.

**Implications for Profession and/or Patient Care:**

Efforts are needed at the European level to strengthen doctoral education in nursing to ensure well‐prepared academic and clinical nurses.

**Impact:**

Findings may support in the development of more cohesive and high‐quality doctoral nursing programs across Europe and inform targeted reforms.

**Reporting Method:**

The rapid review adhered to the Preferred Reporting Items for Systematic Reviews and Meta‐Analysis (PRISMA) guidelines.

**Patient/Public Involvement:**

This study did not include patient or public involvement in its design, conduct or reporting.

**Summary**

- What already is known
  ○ Doctoral nursing education is crucial for advancing nursing science and leadership across clinical, academic and policy contexts.
  ○ Previous reviews have largely focused on US‐based doctoral programs, with limited and outdated evidence regarding the European context.
- What this paper adds
  ○ This study offers an updated synthesis of literature and expert perspectives, providing the most comprehensive overview of doctoral nursing education in Europe to date.
  ○ It identifies seven key challenges and six anticipated changes and highlights the persistent gap between the published evidence and the lived experiences of academic leaders.
- Implications for practice/policy
  ○ Findings offer a robust foundation to inform the strategic reform of doctoral programmes in nursing across Europe, aligned with both research priorities and academic expectations.
  ○ Policymakers, institutions and nursing leaders can use this evidence to promote higher quality, more harmonised doctoral education that addresses system‐wide challenges and fosters future‐ready nurse researchers.

## 1 Introduction

Nurses play a central role in implementing the World Health Organization's (WHO) call for research to address urgent public health challenges, including i.e., poverty‐related diseases, the growing burden of chronic diseases and the promotion of safe health practices. At the same time, both the nursing profession and the discipline emphasise the urgent need for nurses who are able to design and conduct high‐quality research to inform clinical, educational and managerial decisions. Achieving these goals will require substantial investment in nursing education, particularly doctoral education (Teixeira‐Santos et al. 2024).

The value of doctoral education in enhancing nurses' contributions to research is well documented (Dobrowolska, Chruściel, Pilewska‐Kozak, et al. 2021; Teixeira‐Santos et al. 2024). In particular, the Doctor of Philosophy (PhD) as a research‐intensive degree provides nurses with the skills they need to generate, translate and disseminate new knowledge, enabling them to assume leadership roles in academia and the broader professional landscape (Broome et al. 2023; Fisher et al. 2022).

Globally, the development of doctoral education in nursing has been shaped by varying academic traditions and regional contexts. In the United States, doctoral education for nurses began in the early 20th century with the Doctorate of Education (EdD), followed by the Doctorate in Nursing Science (DNSc), and later the PhD. In contrast, access to doctoral education for nurses in Europe emerged later. Due to the absence of nursing‐specific doctoral programs at the time, many European nurses initially pursued doctorates in related fields such as sociology, psychology and education (Dobrowolska, Chruściel, Pilewska‐Kozak, et al. 2021; McKenna 2018; Teixeira‐Santos et al. 2024). Specific doctoral programs for nursing have only existed in Europe since the 1970s, with their expansion accelerated by the Bologna Process reforms. The formal integration of doctoral education into the European academic framework dates to the 2003 Berlin Ministerial Conference, which explicitly recognised the synergy between the European Higher Education Area (EHEA) and the European Research Area (ERA). While the Bologna Declaration of 1999 originally introduced a two‐cycle structure comprising undergraduate and postgraduate studies, the Berlin Communiqué officially acknowledged doctoral education as the third cycle within the EHEA (EHEA 2003).

The traditional doctoral model typically involves 3–4 years of full‐time research (or 5–7 years part‐time), culminating in a dissertation defence. Candidates are usually supervised by two qualified academics, at least one of whom has prior experience in doctoral supervision. While structured coursework is often minimal or absent, candidates may attend specialised seminars on research methodology, academic writing and funding acquisition (McKenna 2018). An alternative to the PhD is the Doctor of Nursing Practice (DNP), which is more common in the United States and, to a lesser extent, the United Kingdom (Teixeira‐Santos et al. 2024). While PhD‐prepared nurses conduct original research to advance nursing science, DNP‐prepared nurses focus on translating existing research into clinical practice through evidence‐based interventions and quality improvement initiatives (Garcia et al. 2025). However, despite the well‐established educational pathways, the most recent reviews (Dobrowolska, Chruściel, Pilewska‐Kozak, et al. 2021; Dobrowolska, Chruściel, Markiewicz, and Palese 2021) found that only 41 studies had examined the state of research related to doctoral programs in nursing through 2019, primarily conducted in the United States (*n* = 26) and to a limited extent in Europe (*n* = 8). The available studies were largely descriptive and documented a lack of international academic collaboration and relative stability in doctoral program structures and processes over time. Most importantly, the studies did not provide a framework for assessing either the short‐ or long‐term outcomes related to program quality and impact. Overall, the results of these reviews (Dobrowolska, Chruściel, Pilewska‐Kozak, et al. 2021; Dobrowolska, Chruściel, Markiewicz, and Palese 2021) show that there is a lack of robust evidence and limited innovative momentum in doctoral education. In addition, no studies were found on the perspectives of academic leaders across Europe. This gap limits the ability to formulate informed strategies that reflect the experiences of those directly involved in the delivery, coordination and advancement of doctoral education in nursing.

A renewed and contextualised review—focused specifically on Europe and incorporating the perspectives of academic leaders—is warranted for several reasons. First, the European higher education landscape is shaped by common frameworks such as the Bologna Process and the EHEA, which promote the comparability and coherence of doctoral programs across countries (Bologna Declaration 1999). Second, European healthcare systems and the role of doctoral education within them share relatively homogeneous characteristics, particularly regarding access, professional autonomy and integration into the broader research ecosystem. Third, synthesising knowledge from a European perspective is particularly valuable for policymakers, academic institutions and professional associations operating within a common legal and cultural framework.

Furthermore, narrowing the geographical focus improves the practical feasibility of a rapid review (Tricco et al. 2017) by limiting the scope of the literature to be assessed, while preserving the relevance of the findings. Moreover, complementing existing evidence with insights from field experts can further support policy development at the European level and potentially reinvigorate doctoral education by contributing to the formulation of an overarching strategic vision. Accordingly, the aim of this study is to offer an innovative and integrative approach to gain a more comprehensive understanding of the current challenges and anticipated developments in doctoral nursing education in Europe—ultimately informing future policy and educational strategies.

### 1.1 Aim

The aim of the study was to capture and summarise the current state of research on doctoral programs in nursing across Europe, to update recent reviews in the field (Dobrowolska, Chruściel, Pilewska‐Kozak, et al. 2021; Dobrowolska, Chruściel, Markiewicz, and Palese 2021), and to complement the available evidence with the insights from experts working in academic and/or doctoral nursing education across Europe (McArthur et al. 2015).

## 2 Methods

### 2.1 Study Designs

Two parallel study designs were used. A rapid review (Tricco et al. 2017) was conducted in June 2025 as a form of knowledge synthesis intended to provide timely information (O'Leary et al. 2017). This followed a seven‐step process: (1) needs assessment and topic selection, (2) study development, (3) literature search, (4) screening and study selection, (5) data extraction, (6) risk of bias assessment and (7) knowledge synthesis. No formal quality assessment of the included studies was performed (Tricco et al. 2017). Concurrently, an online survey was administered to gather expert opinions, either to complement the empirical evidence identified or, in the absence of such evidence, to serve as the best available source of insight (McArthur et al. 2015).

### 2.2 Rapid Review

An initial literature search was conducted following the methods defined in two reviews (Dobrowolska, Chruściel, Pilewska‐Kozak, et al. 2021; Dobrowolska, Chruściel, Markiewicz, and Palese 2021), which included studies published up to December 2019. Subsequently, the same research questions posed in these papers were used to update the review: (a) What is the current state of nursing research related to doctoral programs in Europe? and (b) What are the main topics currently discussed in the available European literature?

**FIGURE 1:**
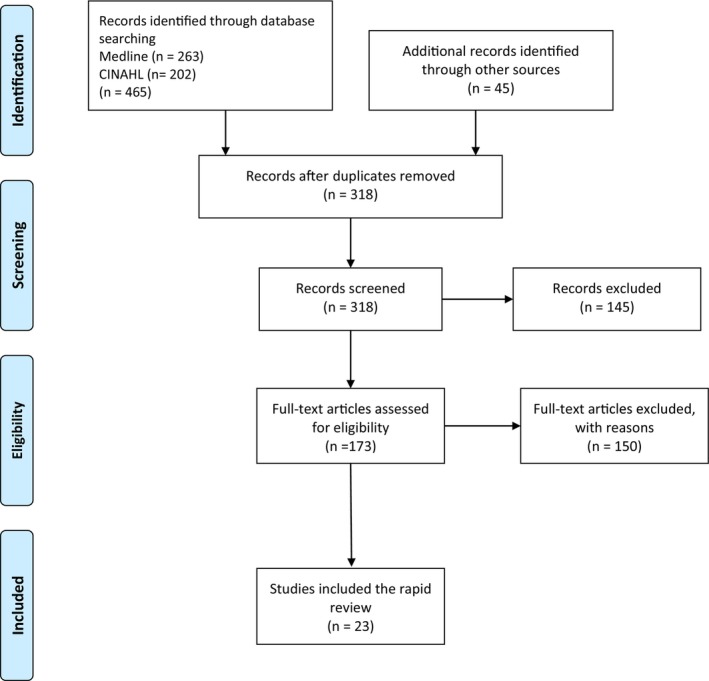
Review flow diagram (Moher et al. 2009). CINAHL, Cumulative Index to Nursing and Allied Health Literature.

The PRISMA (Preferred Reporting Items for Systematic Reviews and Meta‐Analysis) guidelines were followed (Moher et al. 2009) (Table S1). A comprehensive literature search of electronic databases was conducted in May 2025. The Boolean operator AND was used with combinations of the search terms ‘PhD’ and ‘nursing’, in line with previous scoping reviews. The MEDLINE (via PubMed) and Cumulative Index to Nursing and Allied Health Literature (CINAHL) databases were searched for the period January 2020 to June 2025 to match the cut‐off date of the earlier reviews. Ascending citations from the reviews by Dobrowolska, Chruściel, Pilewska‐Kozak, et al. (2021); Dobrowolska, Chruściel, Markiewicz, and Palese (2021) listed in the Scopus database were also searched. Articles were included if they: (a) addressed doctoral nursing education, (b) were written in English, (c) used any study design and (d) were conducted in a European context, or were primary or secondary studies that included data collected in Europe. A total of 510 records were initially identified. After screening and full‐text assessment for eligibility, 23 studies were included in the final review, as shown in the PRISMA flow diagram (Moher et al. 2009) (Figure 1). The following data were extracted from each included study: author(s), year, country (country of origin of the first author or, if available, the country in which the study was conducted), study aim(s), study design, sample and main findings and key recommendations for the future.

### 2.3 Experts' Opinion Survey

One academic nurse from each European country was eligible to participate if they had been involved in the design, implementation, supervision or assessment of doctoral programs in nursing or related disciplines or had published on educational issues or similar topics in recent years and were willing to take part in the study. Initially, the international research team (see authors) compiled a preliminary list of potential participants based on personal networks and existing professional or academic collaborations (purposeful sample). A mailing list was then created and a formal invitation to participate was sent via email. One week later, a reminder was sent to those who had not responded, followed by two additional reminders at one‐week intervals. In cases where no response was received, a second expert was identified using the same strategy—either through personal networks or by reviewing recent publications where the authors' affiliation or research content was relevant to doctoral nursing education. If this second contact did not respond after two reminders, a third expert was selected using the same process. Emails were sent to experts from a total of 31 countries. No response was received from a total of six countries: Bulgaria, Germany, France, Romania, Hungary and Estonia. In summary, opinions were gathered from a total of 26 experts representing 25 countries (including two participants from Spain—one representing the national context and the other specifically from the region of Catalonia). The responding countries included: Albania, Austria, Belgium, Croatia, Cyprus, Czech Republic, Denmark, Finland, Greece, Ireland, Italy, Kosovo, Latvia, Lithuania, Luxembourg, Malta, Netherlands, Norway, Poland, Portugal, Slovakia, Slovenia, Spain and Catalonia, Sweden and Türkiye. Data were collected using an online questionnaire developed via Google Forms. The questionnaire link was included in the invitation letter, which outlined the objectives of the study and described the data collection procedures in June 2025. The questionnaire included open‐ended questions covering three main areas: (a) the demographic and professional profiles of the participants and their roles in doctoral education; (b) perceived challenges or critical issues related to doctoral education in their respective countries; (c) expected or desired changes in the near future to better align doctoral education with expectations. The questions were developed by the research team based on existing literature (Dobrowolska, Chruściel, Pilewska‐Kozak, et al. 2021; Dobrowolska, Chruściel, Markiewicz, and Palese 2021) and underwent content and face validation in two countries (Italy and Poland) to assess clarity and comprehensibility. Additional data were also collected on the characteristics of doctoral programs (e.g., duration, title, topics and degree of internationalisation); however, these data were not analysed in the present study as they fall outside its scope.

### 2.4 Data Analysis

Three steps were adopted for the data analysis.

First, the main findings of the included studies were summarised narratively in the rapid review, following the methodology used by Dobrowolska, Chruściel, Pilewska‐Kozak, et al. (2021); Dobrowolska, Chruściel, Markiewicz, and Palese (2021). These narratives were then presented in summary tables. Second, the expert opinion data collected through the survey were analysed using descriptive statistics to describe the demographic characteristics of the participants. To ensure anonymity, the respondents' countries of origin were grouped into five European regions—Western, Northern, Southern, Central‐Eastern and South‐Eastern Europe/Balkans—based on their geographical orientation. No country‐level analysis was performed, as the aim was to identify broader trends and reforms relevant to Europe as a whole rather than to individual countries. The open‐ended responses were extracted and compiled into a single table. The responses were initially reviewed independently and subsequently consolidated by consensus. After familiarisation with the data, two researchers (BD and GTE) developed a preliminary thematic categorisation based on Robinson (2022) and discussed their interpretations. A third researcher (AP) was then involved in refining and validating the thematic structure. Third, as the study aimed to complement the findings of the rapid review with expert perspectives on current challenges and anticipated developments in doctoral education, a joint narrative synthesis was performed. In this synthesis, qualitative findings from the literature were juxtaposed and integrated with data obtained from the expert survey. An integrative technique, based on corroboration of the literature findings, was employed to support and enhance the experts' contributions. Moreover, participants' insights were particularly valuable in areas where existing literature was limited or absent (Younas and Durante 2023).

### 2.5 Rigour

To ensure methodological rigour, the research team implemented a series of structured strategies. Two subgroups were formed. The first subgroup, consisting of experienced researchers and PhD/Master's students (KB, GD, GM, CM, SC), was responsible for conducting the rapid review. This team adopted the methodology and data analysis strategies previously used in two comprehensive reviews, both of which demonstrated high scientific impact as indicated by citation indices and publication in first quartile (Q1) journals. By adhering to the same methodology, the team aimed to minimise the limitations commonly associated with rapid reviews. To further reduce potential bias, the following strategies were employed: (a) the review team discussed and reached consensus on the inclusion or exclusion of studies at each stage of the review process; (b) three major scientific databases—MEDLINE (via PubMed), CINAHL and Scopus—were systematically searched; (c) data extraction was carried out by a single reviewer and independently verified by at least two other team members (KB, GD, CM, SC). A principal investigator supervised the entire process. The second research subgroup comprised members who were not involved in the rapid review (GTE, BD, GM) and had no knowledge of its results. This approach was adopted to prevent any potential bias in the qualitative data analysis. The survey questions were initially drafted in English (GTE, BD, AP) and then shared with the broader research team for revision. A two‐stage validation process—face validity and content validity—was conducted. To further ensure objectivity and clarity, an external expert (RW), who was not part of the research team, was consulted. Participants were purposefully selected based on their proximity and relevance to the study topic (Patton 2014) to enhance the credibility and depth of the results.

The data analysis was conducted independently and then consolidated. Three researchers (AP, BD, GTE) worked across multiple sessions and rounds. The remaining members of the research team subsequently reviewed the extracted and synthesised data to validate the findings.

### 2.6 Ethical Considerations

For the Rapid Review, ethical approval was not required. According to the national rules of the leading countries (see authors), the research protocol was not eligible for ethical approval by the Ethics Committee or the Institutional Review Board. The experts participating in the opinion survey were informed in advance about the study's aims and procedures. Data confidentiality was maintained throughout the research process, and participant anonymity was safeguarded by avoiding country‐level analyses. To further protect participant identities, geographic data was aggregated into five broader European regions. The entire process adhered to the ethical principles of the Declaration of Helsinki, and participant burden was minimised by limiting follow‐up communications to two reminders. Reported challenges were analysed without linking responses to specific countries, thereby ensuring both anonymity and confidentiality.

## 3 Results

### 3.1 Rapid Review

As shown in Figure 1 and the Table S2, a total of 23 studies were identified, originating from or affiliated with institutions in 18 European countries. These included: Bulgaria (*n* = 1; Taneva et al. 2023); Finland (*n* = 3; Tikkanen et al. 2025; Van Dongen, Hafsteinsdottir, et al. 2024; Van Dongen, Suidman, et al. 2024); France (*n* = 1; Met et al. 2022); Italy (*n* = 1; Ottonello et al. 2024); Italy/Germany (*n* = 1; Zerbe 2025); Portugal (*n* = 1; Lino et al. 2022); Slovenia (*n* = 1; Skela‐Savič et al. 2020); Spain (*n* = 1; Dalfó‐Pibernat et al. 2020); Sweden (*n* = 2; Holmberg 2024; Orton et al. 2022); the Netherlands (*n* = 3; Hafsteinsdóttir et al. 2025; Sterkenburg et al. 2025; Van Dongen and Hafsteinsdóttir 2022); and the United Kingdom (*n* = 3; Henshaw et al. 2025; Sanders et al. 2022; Wong et al. 2025). The remaining five publications were review articles that included European data but were not country‐specific (Cleary et al. 2023; McBride‐Henry et al. 2024; Negarandeh and Khoshkesht 2022; Stolldorf et al. 2022; Tyndall et al. 2021). The studies were published between 2020 (*n* = 2; Dalfó‐Pibernat et al. 2020; Skela‐Savič et al. 2020) and 2025 (*n* = 6; Hafsteinsdóttir et al. 2025; Henshaw et al. 2025; Sterkenburg et al. 2025; Tikkanen et al. 2025; Wong et al. 2025; Zerbe 2025). A total of eight reviews were identified, including one systematic review (Orton et al. 2022), three narrative reviews (Cleary et al. 2023; Negarandeh and Khoshkesht 2022; Wong et al. 2025), one literature review (Stolldorf et al. 2022), two integrative reviews (Tyndall et al. 2021; Van Dongen, Hafsteinsdottir, et al. 2024), and one exploratory study conducted in three phases—scoping review, document analysis and thematic analysis (McBride‐Henry et al. 2024). Four discussion‐type papers were included: three discussion papers (Henshaw et al. 2025; Sanders et al. 2022; Zerbe 2025) and one discursive paper (Ottonello et al. 2024). Three qualitative studies were reported: a qualitative descriptive study using semi‐structured interviews and thematic analysis (Van Dongen and Hafsteinsdóttir 2022), a descriptive exploratory qualitative study (Lino et al. 2022), and one survey study (Tikkanen et al. 2025). Two experimental studies were identified: a pre‐test–post‐test survey (Hafsteinsdóttir et al. 2025) and a pre‐post test study with a convergent mixed methods design (Van Dongen, Suidman, et al. 2024). Two studies employed mixed methods: a two‐stage mixed methods design (Met et al. 2022) and a combination of a descriptive cross‐sectional study with qualitative elements (Skela‐Savič et al. 2020). Additional study types included a cross‐sectional observational study (Dalfó‐Pibernat et al. 2020), a Delphi study (Sterkenburg et al. 2025), a bibliometric and mapping analysis (Holmberg 2024), and a comparative study involving research articles, internet data and emails (Taneva et al. 2023).

The number of participants ranged from two PhD nurses (Ottonello et al. 2024) to 561 PhD supervisors (Tikkanen et al. 2025); for the reviews, the number of included studies ranged from nine (Stolldorf et al. 2022) to 7213 (Holmberg 2024). The main participants were nurses with a PhD degree (Hafsteinsdóttir et al. 2025; Ottonello et al. 2024; Sterkenburg et al. 2025; Van Dongen and Hafsteinsdóttir 2022; Van Dongen, Suidman, et al. 2024). Additionally, PhD students (Skela‐Savič et al. 2020; Van Dongen, Suidman, et al. 2024), PhD nursing professors (Lino et al. 2022) and multidisciplinary PhD supervisors (Tikkanen et al. 2025) were included. Moreover, primary care nurses with a PhD (Dalfó‐Pibernat et al. 2020), healthcare managers and senior nurses (Met et al. 2022), and a multidisciplinary group (Sanders et al. 2022) were also included.

### 3.2 Experts' Opinion Survey

The study included 26 participants, mainly from Southern Europe (11.5%) and female (69.2%). Their ages ranged from 31 to 69 years, with a mean age of 52.8 years (SD = 10.3). Regarding the doctoral programs available in their institutions or countries, 53.8% indicated that a doctoral degree was available in nursing or nursing sciences, followed by 30.8% in health sciences and 19.2% in multidisciplinary fields such as medicine, public health, clinical or biomedical sciences. Additionally, 3.8% indicated the availability of a doctorate in nursing management, while another 3.8% stated that no PhD program in nursing exists in their country. Participants had been involved in doctoral programs for an average of 11.5 years (SD = 8.9). Based on experience levels, 34.6% were categorised as early‐stage participants (0–5 years), 42.3% as mid‐career participants (6–15 years), and 23.1% as senior participants with ≥ 16 years of involvement. Regarding doctoral supervision, participants had supervised an average of 9.6 PhD students throughout their academic careers (SD = 10.3). Four participants had never supervised a doctoral student, while three had supervised more than 21 students (Table 1).

**TABLE 1:** Population characteristics (*n* = 26).

Variables	*n* (%)
Gender	
Female	18 (69.2)
Male	8 (30.8)
Age groups (*M* = 52.8, SD = 10.3)	
30–44 years	6 (23.1)
45–59 years	11 (42.3)
≥ 60 years	9 (34.6)
Name of the PhD program available in your institution/country,*	
PhD in Nursing/Nursing Sciences	14 (53.8)
PhD in Health Sciences	8 (30.8)
PhD in Medicine/Public Health/Clinical/Biomedical Sciences	5 (19.2)
PhD in Nursing Management	1 (3.8)
No PhD program in nursing field	1 (3.8)
How many years have you been involved in the PhD program? (*M* = 11.5, SD = 8.9)	
0–5 years	9 (34.6)
6–15 years	11 (42.3)
≥ 16 years	6 (23.1)
How many PhD students have you supervised in your academic life? (*M* = 9.6, SD = 10.3)	
None	4 (15.4)
1–10 students	12 (46.2)
11–20 students	7 (26.9)
≥ 21 students	3 (11.5)
Region	
Southeastern Europe/Balkans	
Albania, Kosovo, Türkiye	3 (11.5)
Western Europe	
Austria, Belgium, Ireland, Luxembourg, Netherlands	5 (19.2)
Northern Europe	
Denmark, Finland, Latvia, Norway, Sweden	5 (19.2)
Central‐ and Eastern‐Europe	
Croatia, Czech Republic, Lithuania, Poland, Slovakia, Slovenia	6 (23.1)
Southern Europe	
Cyprus, Greece, Italy, Malta, Portugal, Spain, Spain Catalonia	7 (27.0)

*Note:* *, more than one answer was possible.

Abbreviations: *M*, mean; *n*, number; PhD, Doctor of Philosophy; SD, standard deviation.

#### 3.2.1 Integration of the Findings: Challenges

As shown in Table 2 (see also Table S2), seven key challenges in doctoral nursing education were identified across different levels: institutional and structural; supervision‐related; candidate‐related; research process and output; professional and career‐related; international collaboration; and paradigm‐related. Some of these challenges have already been documented in the literature as highlighted in the rapid review, while others remain underrecognized or have yet to be fully acknowledged. This may partly explain why not all countries in the EHEA currently offer doctoral programs in nursing. For instance, such programs are absent in Estonia, Latvia and France (Taneva et al. 2023).

**TABLE 2:** Challenges of doctorate programs across Europe: experts' opinion and rapid review findings integration.

No	Main challenges	Experts' opinion	Rapid review
1	Institutional and Structural challenges	Poor funding and financial support Lack of or poor financial support for PhD nursing students Limited access to research grants Lower funding opportunities than for other disciplines No national programs to promote PhD in nursing	Met et al. (2022) Skela‐Savič et al. (2020) Taneva et al. (2023) Zerbe (2025)
		Lack of dedicated infrastructure Lack of independent nursing PhD programs Inadequate research infrastructure Insufficient institutional support Lack of enough places for PhD applicants	
		Program inconsistency and fragmentation Differences in PhD programs across Europe (variation in program length, format and requirements) Lack of mutual recognition between countries	
		Limited national support and visibility Nursing is not prioritised in national research agendas Biomedical paradigm dominance Low visibility of nursing research compared to other disciplines	Met et al. (2022)
2	Supervision‐related challenges	Shortage of qualified supervisors Limited access to experienced nurse supervisors/mentors	Cleary et al. (2023)
		Low supervision quality Supervisors lacking research experience or specific competencies Poor motivation among supervisors due to lack of support or recognition	Tikkanen et al. (2025)
		Lack of support provided to PhD supervisors no incentives or institutional recognition for supervision	
3	Candidate‐related challenges	Competency gaps Low research competence of applicants Lack of appropriate candidates' selection or assessment Inconsistent levels of research experience	
		Low motivation and interest PhD pursued only as a formal requirement (e.g., for employment) Decreasing interest in PhD programs among master's graduates in nursing	
		Personal and professional strain High clinical workload and limited time for research Work‐family balance issues Struggles balancing PhD with full‐time employment	
		Limited career incentives Low salary prospects Minimal advancement opportunities Weak linkage between PhD and leadership or policy influence	
4	Research process and output challenges	Barriers to conducting research Difficulty in recruiting research participants Ethical and legal approval issues Insufficient support in data collection	Stolldorf et al. (2022)
		Publishing and dissemination barriers Limited capability to publish in high‐impact journals Pressure to publish to complete PhD Poor integration of innovative methods or topic	Holmberg (2024) Tyndall et al. (2021) Wong et al. (2025)
		Methodological challenges Difficulty in applying or justifying nursing‐specific methodologies Lack of theoretical frameworks adapted to nursing research	Tyndall et al. (2021)
5	Professional and career‐related challenges	Poor recognition and unclear career pathways Limited professional roles for PhD‐prepared nurses Lack of integration of research into clinical/managerial roles No defined career ladder for nurses with PhD Lack of role models or recognition in clinical settings No guarantee of employment in academia or clinical settings Lack of clinical relevance or utility of PhD title in practice	Dalfó‐Pibernat et al. (2020) Hafsteinsdóttir et al. (2025) Henshaw et al. (2025) McBride‐Henry et al. (2024) Negarandeh and Khoshkesht (2022) Orton et al. (2022) Ottonello et al. (2024) Sanders et al. (2022) Sterkenburg et al. (2025) Stolldorf et al. (2022) Van Dongen and Hafsteinsdóttir (2022), Van Dongen, Hafsteinsdottir, et al. (2024), Van Dongen, Suidman, et al. (2024)
6	International collaboration challenges	Barriers to cross‐national exchange Differences in PhD programs in nursing across countries Limited international mobility of PhD students (e.g., because of family or work‐related issues) Weak collaboration across borders of PhD students in nursing	Lino et al. (2022)
7	Paradigm challenges	Weak/short academic tradition in nursing Short tradition of academic nursing in some countries Biomedical paradigm marginalising nursing science Lack of recognition in broader research teams	Met et al. (2022)

Abbreviation: PhD, Doctor of Philosophy.

##### 3.2.1.1 Institutional and Structural Challenges

The experts reported several institutional and structural barriers, including a lack of resources for both students and institutions, insufficient infrastructure to support independent doctoral programs and limited availability of academic positions. This may partly explain why not all countries in the EHEA currently offer doctoral programs in nursing. For example, such programs are absent in Estonia, Latvia and France (Taneva et al. 2023). Where doctoral programs do exist (Skela‐Savič et al. 2020; Taneva et al. 2023; Zerbe 2025), differences in their structural and organisational characteristics have been documented, indicating that structural barriers are more pronounced in countries lacking formal programs. In these contexts, nursing doctoral education often suffers from low visibility and recognition, a lack of clearly defined academic roles and positions, and limited integration into clinical and research environments. Furthermore, discrepancies in the duration, formats and requirements of doctoral programs have been found to hinder mutual recognition across countries (Met et al. 2022).

##### 3.2.1.2 Supervision‐Related Challenges

Qualified supervisors were reported by experts as not always available, and the quality of supervision is sometimes compromised due to a lack of competence or motivation, often linked to limited institutional recognition of the mentoring role (e.g., insufficient protected time). Overall, the mentor–mentee relationship is regarded as essential to the success of doctoral studies. Cleary et al. (2023) examined mentoring experiences in doctoral nursing programs and emphasised the importance of compatibility, personal support and regular interaction. Effective mentoring fosters the development of research skills, academic writing and critical thinking, which ultimately facilitates dissertation completion and academic advancement. Furthermore, Cleary et al. (2023) advocate for the integration of structured mentoring models into doctoral programs, particularly where such systems are not yet established.

However, gaps remain in faculty mentor training, highlighting the need for formal training, institutional incentives and co‐mentoring models based on interdisciplinary and networked approaches. Despite these recommendations, persistent challenges include limited mentor availability, inadequate preparation, lack of commitment and burnout among mentors (Tikkanen et al. 2025).

##### 3.2.1.3 Candidate‐Related Challenges

According to experts, candidates face various difficulties, particularly regarding their initial research skills, which are often linked to the selection criteria for admission to the program. It was also noted that some candidates are primarily motivated by career advancement rather than a genuine desire to contribute to research. This lack of intrinsic motivation may partly explain the low number of applicants coming from master's programs. Additionally, work‐life balance significantly limits the time and energy that candidates can devote to their studies and research.

##### 3.2.1.4 Research Process and Output Challenges

The path to a doctorate is often associated with challenges related to conducting research and achieving expected outcomes. According to experts, difficulties frequently arise in recruiting study participants, obtaining ethical approval and addressing legal considerations such as data protection. Students face significant pressure to publish their work but often lack the necessary skills, resources or support to meet the high standards for scientific publications. Consequently, dissemination of research findings becomes a major obstacle. Furthermore, doctoral research in nursing tends to be traditional and involves limited use of innovative methods. This may stem from challenges in applying or justifying novel approaches or anchoring research within a robust theoretical or conceptual framework. Tyndall et al. (2021) examined threshold concepts in doctoral education across the stages of development, implementation and dissemination. They identified key academic challenges and proposed strategies to support student progression, particularly in writing and research design.

Contributing factors included writing development, community presence and faculty influence. Group discussion, structured reading, academic writing and “talking to think” were identified as effective strategies to support conceptual development. Similarly, Wong et al. (2025) emphasised the central role of academic writing in doctoral education and identified six evidence‐based themes—facilitation models, writing barriers, accountability and productivity, group identity and collegiality, peer review and behaviour change—to support institutions in improving doctoral students' writing skills and productivity. However, in some countries, such as Sweden, high researcher productivity has been documented (Holmberg 2024), mainly attributed to collaboration among universities, hospitals and other countries. Scientific productivity, number of presentations, number of publications, peer review activities and funded and/or submitted grants have been considered in alumni surveys to assess outcomes after graduation (Stolldorf et al. 2022).

##### 3.2.1.5 Professional and Career‐Related Challenges

The professional development of post‐doctoral nurses remains a significant and complex challenge across Europe—according to experts' report. Despite the growing importance of doctoral education in nursing, the doctorate is still only partially recognised, especially in the clinical context. There are insufficient positions in both academic and clinical research, and integrating research responsibilities into clinical or managerial roles remains difficult. Promotion opportunities are still limited, and the lack of established role models in the clinical settings poses a barrier for newly graduated doctors. Additionally, job security is often uncertain, and the value of a doctorate—particularly in clinical practice—is sometimes questioned. Nevertheless, evidence indicates that doctoral education can enhance nurses' confidence and competence in managing complex clinical situations (Dalfó‐Pibernat et al. 2020). These challenges are exacerbated by inconsistencies in the formal definition of roles and responsibilities for postgraduate nurses (Negarandeh and Khoshkesht 2022) and the limited availability of structured career opportunities within healthcare systems (Orton et al. 2022; Ottonello et al. 2024). Several studies (Dalfó‐Pibernat et al. 2020; Negarandeh and Khoshkesht 2022; Orton et al. 2022; Ottonello et al. 2024; Sanders et al. 2022; Van Dongen and Hafsteinsdóttir 2022) have examined how nurses with PhDs are integrated (or not) into healthcare organisations. Their findings suggest that PhD nurses can make an important contribution to evidence‐based practice, lead clinical innovation projects and promote a research‐oriented culture in healthcare organisations. However, these contributions are often hindered by structural constraints, the tension between clinical and academic dual roles and inadequate institutional support. A recent discussion paper by Henshaw et al. (2025) confirms that the number of nurses with a PhD remains relatively low. To address this gap, various models have been proposed to support academic and clinical career development. These include structured leadership and mentoring programs aimed at enhancing self‐efficacy, research readiness and the capacity to assume academic or leadership roles (e.g., the Leadership Mentoring in Nursing Research and Nurse‐Lead initiatives) (Hafsteinsdóttir et al. 2025; Van Dongen and Hafsteinsdóttir 2022; Van Dongen, Suidman, et al. 2024). Van Dongen and Hafsteinsdóttir (2022) also noted that nurses' career advancement after graduation often depends on personal motivation, long‐term planning and a strong commitment to the profession and highlighted the general lack of systematic data on the career success of this group (Van Dongen, Suidman, et al. 2024). To address these issues, coordinated strategies involving universities, healthcare employers and professional organisations are needed to encourage earlier engagement in doctoral education and to establish viable career pathways after graduation. Although all doctoral pathways (e.g., PhD and PhD by publication) are scientifically valid, concerns remain regarding their inconsistent recognition across different systems. In the UK, for example, the National Institute for Health and Care Research (NIHR) does not consistently accept professional doctorates for its funding programs, despite their academic equivalence. Nonetheless, NIHR initiatives to promote nursing research through fellowships and career development programs remain valuable, as they support both individual career progression and improvements in patient care outcomes (Henshaw et al. 2025).

To address the lack of a standardised framework for evaluating graduate outcomes, McBride‐Henry et al. (2024) proposed a comprehensive model comprising five domains to assess the personal, academic and professional development of PDPN graduates. To facilitate the evaluation of postdoctoral nurses' competencies, Sterkenburg et al. (2025) developed the Postdoctoral Nurses Competence Scale (PNCS), a validated self‐assessment instrument with 13 items. This instrument has demonstrated strong content validity and offers a promising approach for assessing the competencies of postdoctoral nurses in both clinical and academic settings. In the absence of such frameworks, graduate outcomes are commonly tracked using indicators such as employment status, scholarly productivity, leadership roles, publications, research funding and self‐perceived growth in knowledge and skills (Stolldorf et al. 2022). While the five‐domain framework of McBride‐Henry et al. (2024) and the PNCS instrument of Sterkenburg et al. (2025) represent significant advancements, their integration into doctoral program curricula and institutional evaluation strategies remains limited.

##### 3.2.1.6 International Collaboration Challenges

Our experts report that internationalisation remains a major challenge due to inconsistencies between doctoral programs, individual limitations of candidates and weak intra‐European cooperation. The contribution of the Bologna Process to academic‐professional mobility in nursing was investigated by Lino et al. (2022) in a qualitative, descriptive study involving senior Portuguese nurses. The results revealed three key dimensions. First, academic mobility and internationalisation were perceived as strategic tools for strengthening the European economic bloc by enhancing competitiveness, knowledge exchange and professional development. Second, mobility contributed to the consolidation of a common European identity, fostering cultural integration, reflexivity and both personal and professional transformation. Third, the introduction of the Diploma Supplement emerged as a crucial mechanism for promoting comparability and mutual recognition of qualifications, thus facilitating both academic and professional mobility. Despite the positive developments, the study also highlighted ongoing challenges such as disparities in education systems, limited internationalised curriculum content and language barriers.

##### 3.2.1.7 Paradigm Challenges

Finally, the experts also raised concerns regarding research paradigms in nursing. The lack of a well‐established research tradition, the marginalisation of nursing within academic institutions in favour of other disciplines and the limited recognition of nursing research all contribute to the weak status of nursing doctorates within the broader academic community. According to the findings of a recent rapid review, doctoral programs in nursing often remain invisible due to the dominant position of medicine in the national academic landscape (Met et al. 2022).

#### 3.2.2 Integration of the Findings: Changes and Recommendations

As shown in Table 3 (see also Table S2), six anticipated changes have been identified as priorities for the advancement of doctoral nursing education across Europe.

**TABLE 3:** Changes and recommendations: experts' opinion and rapid review findings integration.

No	Main area	Experts' opinion	Rapid review
1	Structural and policy	Program design and structure Develop independent PhD programs in nursing with discipline‐specific focus Support both traditional academic and clinical doctorate pathways Offer flexible, modular, hybrid or part‐time study formats Include personalised study plans and microcredentials Embed implementation science, leadership and digital competencies (e.g., AI) Create Europe‐wide frameworks for quality, credits and supervision standards Promote joint degrees, cotutelle programs and doctoral networks across countries	Lino et al. (2022) McBride‐Henry et al. (2024) Met et al. (2022) Stolldorf et al. (2022) Tyndall et al. (2021) Van Dongen, Hafsteinsdottir, et al. (2024) Wong et al. (2025)
		Curriculum modernisation Emphasise research over didactic education Ensure a strong foundation in nursing research methods Foster interdisciplinary and transdisciplinary learning while maintaining nursing identity Include topics like health equity, societal impact and open science, AI, etc	Negarandeh and Khoshkesht (2022) Skela‐Savič et al. (2020) Tyndall et al. (2021) Van Dongen and Hafsteinsdóttir (2022) Van Dongen, Suidman, et al. (2024) Wong et al. (2025) Zerbe (2025)
		Governance and quality assurance Conduct regular program reviews/evaluation Establish external committees to evaluate PhD theses/and PhD programs Implement benchmarking of nursing PhD programs globally Develop clear agendas and research priorities aligned with health system needs	Lino et al. (2022) McBride‐Henry et al. (2024) Met et al. (2022) Stolldorf et al. (2022) Van Dongen, Suidman, et al. (2024)
2	Supervision and mentorship	Supervisors development Establish criteria for supervisor selection and performance evaluations Encourage junior academics to co‐supervise with experienced faculty Provide training on mentorship best practices Offer workload compensation and incentives for supervisors Promote co‐supervision models combining diverse expertise	Hafsteinsdóttir et al. (2025) Tikkanen et al. (2025) Zerbe (2025)
		Mentorship and career support Develop structured mentorship programs Track career progression and professional impact Provide career planning and postdoctoral development pathways	Cleary et al. (2023) Hafsteinsdóttir et al. (2025) Sanders et al. (2022) Sterkenburg et al. (2025) Van Dongen and Hafsteinsdóttir (2022)
3	Candidate recruitment, retention and support	Recruitment strategies Improve selection criteria for PhD candidates Motivate strong master's graduates to pursue PhDs Encourage diverse academic and professional backgrounds Actively promote career opportunities in research and academia	Henshaw et al. (2025) Orton et al. (2022) Van Dongen, Suidman, et al. (2024) Zerbe (2025)
		Retention strategies Offer scholarships, research assistance and funded study positions Design flexible study models for working professionals Provide supportive learning environments with academic communities	Sanders et al. (2022) Sterkenburg et al. (2025) Tikkanen et al. (2025)
4	Financial and institutional support	Funding and resources Increase research funding and financial aid for students Secure funding for large‐scale nursing‐led studies Provide financial support from clinical institutions for enrolled nurses employed in clinics create research infrastructure: libraries, databases, labs, statistical support	Sanders et al. (2022) Stolldorf et al. (2022)
		Workplace and institutional support Establish clinical‐academic integration pathways Offer funded research time and shared PhD projects with hospitals Support research in nursing through national research agencies Encourage collaboration between academia and practice	Met et al. (2022) Negarandeh and Khoshkesht (2022) Ottonello et al. (2024)
5	Professional development and career recognition	Career pathways and recognition Build clear career trajectories for PhD‐prepared nurses Ensure formal recognition in clinical, managerial and academic roles Offer career incentives inside and outside academia Develop dual clinical‐research roles and APN/CNS leadership integration	McBride‐Henry et al. (2024) Met et al. (2022) Negarandeh and Khoshkesht (2022) Orton et al. (2022) Ottonello et al. (2024) Sanders et al. (2022) Sterkenburg et al. (2025) Van Dongen, Hafsteinsdottir, et al. (2024)
		Nursing leadership and visibility Recognise nurses with PhD as leaders in education, policy and research Expand international mobility and leadership training	Hafsteinsdóttir et al. (2025) Orton et al. (2022) Stolldorf et al. (2022) Van Dongen, Hafsteinsdottir, et al. (2024)
6	Collaboration and internationalisation	Interdisciplinary and interprofessional cooperation Promote interdisciplinary faculty involvement in PhD programs Encourage cross‐sector collaboration in health, data science and policy Ensure nursing remains central in interdisciplinary work	Lino et al. (2022) McBride‐Henry et al. (2024) Ottonello et al. (2024) Wong et al. (2025)
		International collaboration and mobility Support international students access to PhD programs Foster international collaboration and exchange Strengthen European doctoral networks and joint projects Engage international experts in PhD programs/mentoring and education	Hafsteinsdóttir et al. (2025) Holmberg (2024) Lino et al. (2022) McBride‐Henry et al. (2024) Taneva et al. (2023)

Abbreviations: APN, Advanced Practitioner Nurse; CNS, Clinical Nurse Specialist; PhD, Doctor of Philosophy.

The first relates to the overall structure of doctoral programs and the need for reform of program design, curriculum content and quality control. In general, a modernisation of curricula is expected, including the integration of contemporary and relevant topics and the introduction of more flexible models that are aligned with European standards, emphasising clinical relevance and promoting multidisciplinary approaches. These structural improvements should be supported by transparent accreditation systems and rigorous quality assurance mechanisms that promote benchmarking and support transnational dialogue. These recommendations are also based on the findings of the studies included in the rapid review (Lino et al. 2022; McBride‐Henry et al. 2024; Met et al. 2022; Negarandeh and Khoshkesht 2022; Skela‐Savič et al. 2020; Stolldorf et al. 2022; Tyndall et al. 2021; Van Dongen and Hafsteinsdóttir 2022; Van Dongen, Hafsteinsdottir, et al. 2024; Wong et al. 2025; Zerbe 2025), with the exception of proposals relating to the provision of flexible study formats and the development of research agendas tailored to the needs of the healthcare system.

A second area concerns the development and support of doctoral supervision and mentorship. There is an urgent need to professionalise supervision by introducing clear admission criteria, structured training pathways and the early involvement of early career researchers in co‐supervision. Supervisors should be adequately supported by institutions, formally recognised and appreciated for their academic supervision work. The recommendations that emerged from the rapid review were broadly in line with these points (Cleary et al. 2023; Hafsteinsdóttir et al. 2025; Sanders et al. 2022; Sterkenburg et al. 2025; Tikkanen et al. 2025; Van Dongen and Hafsteinsdóttir 2022; Zerbe 2025), although the authors did not explicitly address the provision of remuneration and incentives for supervisors or the promotion of co‐supervision models that integrate different levels of expertise. The approach to selecting and supporting doctoral students needs to be further modified. Robust recruitment mechanisms are essential to identify promising candidates early and nurture their research potential from the outset. Retention strategies must ensure that doctoral students receive academic, financial and infrastructural support in a high‐quality environment that fosters research and intellectual development. The recommendations from the review are also consistent with these needs (Henshaw et al. 2025; Orton et al. 2022; Sanders et al. 2022; Sterkenburg et al. 2025; Tikkanen et al. 2025; Van Dongen, Suidman, et al. 2024; Zerbe 2025). However, the studies did not include specific recommendations for improving the selection criteria for doctoral students or for designing flexible study models tailored to the needs of working professionals.

Another important area of reform concerns sustainability and funding. Investment should not only be directed towards strengthening research infrastructures, but also towards direct financial support for students through scholarships and access to substantial research funding. Specific strategies need to be implemented to facilitate the participation of clinical nurses in doctoral education—especially those working in hospitals or communities where access to academic programs remains limited. These issues were addressed in the rapid review (Met et al. 2022; Negarandeh and Khoshkesht 2022; Ottonello et al. 2024; Sanders et al. 2022; Stolldorf et al. 2022). However, the studies did not include recommendations for increasing research funding overall, expanding financial support for students or encouraging clinical institutions to financially support enrolled clinical nurses.

In addition, changes are also expected in career development. Clearly defined and formalised career paths for postgraduate nurses need to be created, accompanied by appropriate incentives and institutional support—in particular, recognition of their dual role as clinician and researcher. This recognition should extend to both healthcare and academic systems to ensure that nurses with PhDs can make a meaningful contribution to innovation, evidence‐based practice and leadership. Improving their national and international visibility is also important to enhance their influence and professional legitimacy. The recommendations emerging from the rapid review are closely aligned with these goals (Hafsteinsdóttir et al. 2025; McBride‐Henry et al. 2024; Met et al. 2022; Negarandeh and Khoshkesht 2022; Orton et al. 2022; Ottonello et al. 2024; Sanders et al. 2022; Sterkenburg et al. 2025; Stolldorf et al. 2022; Van Dongen, Hafsteinsdottir, et al. 2024).

Finally, more emphasis should be placed on promoting interdisciplinary, interprofessional and international cooperation. Strengthening collaboration not only across professional and sectoral boundaries but also across national borders is essential for improving the quality, relevance and mobility of doctoral education in nursing. These forms of collaboration are important not only for the academic development of doctoral students and their supervisors but also for the alignment of nursing education with the wider European research and policy agenda. The recommendations of the rapid review are consistent with these priorities (Hafsteinsdóttir et al. 2025; Holmberg 2024; Lino et al. 2022; McBride‐Henry et al. 2024; Ottonello et al. 2024; Taneva et al. 2023; Wong et al. 2025). However, the studies did not address the need to ensure that nursing retains a central role in interdisciplinary initiatives.

## 4 Discussion

### 4.1 Rapid Review

A rapid review was conducted to update the most recent synthesis in this field (Dobrowolska, Chruściel, Pilewska‐Kozak, et al. 2021; Dobrowolska, Chruściel, Markiewicz, and Palese 2021). The production of studies amounts to about four to five publications per year, originating from a limited number of European countries, with increasing contributions from multi‐country regions within and outside Europe. This trend indicates a stable reflection on the role of doctoral education in nursing, considering that the previous review included about 40 studies over 10 years (Dobrowolska, Chruściel, Pilewska‐Kozak, et al. 2021; Dobrowolska, Chruściel, Markiewicz, and Palese 2021).

Most of the available publications are from Northern European countries and the United Kingdom and consist mainly of review articles and discussion or discourse pieces. The main thematic foci of these studies include mentoring and supervision (e.g., Cleary et al. 2023), curricula and educational content (Skela‐Savič et al. 2020), doctoral programs and models (e.g., Henshaw et al. 2025), outcomes and impact of doctoral programs (e.g., McBride‐Henry et al. 2024), career trajectories and roles of doctoral nurses (e.g., Van Dongen, Hafsteinsdottir, et al. 2024), postdoctoral career development and academia (e.g., Sanders et al. 2022), evaluation methods and metrics (e.g., Stolldorf et al. 2022), internationalisation and education policy (Lino et al. 2022), and disciplinary and regional research trends (e.g., Holmberg 2024).

Overall, doctoral programs in nursing have different formats and structures in European countries (Zerbe 2025), which is consistent with the findings of Dobrowolska, Chruściel, Pilewska‐Kozak, et al. (2021). Even 23 years after the adoption of the Bologna Process, there are still considerable differences in nursing education. While significant progress has been made in harmonising education systems and identifying points of convergence, notable differences continue to hinder full alignment with the objectives of the Bologna Process and the broader vision of the EHEA (Taneva et al. 2023). Although the Bologna Process has promoted improvements in professional mobility and identity formation, mobility opportunities remain unevenly distributed (Lino et al. 2022). Furthermore, while there are multiple doctoral pathways (PhD, DProf, PhD by publication), the recognition of professional doctorates remains inconsistent across countries, as is evident in the UK (Henshaw et al. 2025). These inconsistencies underline the need for further efforts to harmonise doctoral education in nursing in Europe in terms of structure, content and formal recognition.

### 4.2 Experts' Opinion Survey

As far as we are aware, there is no study that has captured the views of European leaders on the issues and reforms needed to strengthen doctoral education based on their direct experience. Participants from Bulgaria, Estonia, France, Germany, Hungary and Romania were not reached, suggesting that these countries need to be included in the future. The participants were nurses with research experience. Most of them were already involved in doctoral programmes, some even at an early stage, and had not yet supervised a doctoral student. Nevertheless, their professional background and expertise qualified them as valuable informants, which is also reflected in the number of students supervised.

#### 4.2.1 Findings Integration: The Challenges

In our survey, most of the participating experts came from Southern Europe, while the analysed literature was mainly produced in Northern Europe. Some of the challenges identified overlapped with findings that had already been documented in the last 5 years and therefore served to confirm them (e.g., issues of professional development and postdoctoral careers), while others proved to be new and not yet sufficiently researched (such as the lack of skills of candidates). Gathering practical, lived experiences provides a more vivid and up‐to‐date understanding of current challenges. Although we have only considered the most recent literature from the last 5 years, due to the inevitable time lag in academic publications, reviews may not always reflect the current context and thus inform decision‐making to a limited extent. Furthermore, certain perceptions may remain undocumented—possibly because they are too entrenched in everyday practice or have not yet been deemed worthy of dissemination through formal research.

Overall, doctoral programs in nursing across Europe are still very fragile structurally and institutionally. Experts reported a lack of financial and infrastructural support, as well as unclear frameworks that require greater alignment and a shared strategic vision. The limited visibility of nursing, particularly compared to other academic disciplines, further weakens investment in the specialty, a challenge that has been documented in the literature (e.g., Taneva et al. 2023). This weakness also extends to supervision, which is often inadequately supported. Effective supervision requires time, expertise and motivation—resources that are not always available (Cleary et al. 2023).

Embarking on a doctoral program also requires a set of basic research skills that many candidates do not fully possess. This emphasises the need for increased preparation at the master's level, which usually serves as an entry point into doctoral education. In addition, careful time management is crucial to allow for adequate study and to create a healthy balance with professional and personal commitments. The provision of bursaries or other targeted support would be particularly beneficial for those with family commitments and would contribute to more equitable participation. This challenge appears to be emerging and requires specific strategies to ensure that candidates are also suitable for doctoral study and that the pathway leads to success.

An important unresolved issue remains how to assess doctoral outcomes and support career development—areas that still receive too little attention and resources, particularly in the clinical context. Internationalisation is also limited, and concerns remain about the marginalisation of nursing research and its perceived lack of relevance. The overall findings are consistent with the previous review (Dobrowolska, Chruściel, Pilewska‐Kozak, et al. 2021), which emphasises the need for agreed outcome measures that assess not only doctoral processes but also short‐ and long‐term outcomes, including professional development and satisfaction. However, while some of the challenges cited by the experts—such as professional development and career progression—have been widely documented, others—such as candidate preparation and fragility of supervision—have received little attention in the recent literature. This suggests that the academic focus tends to concentrate on postgraduate outcomes, while the fundamental dyad—candidate and supervisor—that is central to the development of research skills is clearly recognised in everyday practice but remains under‐represented in academic discourse.

#### 4.2.2 Findings Integration: Changes and Recommendations

The experts outlined a differentiated map of the areas in which changes are to be expected—a map that could serve as the basis for a comprehensive European strategy for the development of doctoral education in nursing. Within this map, three key dimensions of interest emerged. Firstly, some of the recommendations are directly related to challenges already experienced in practice and described in the literature. These include, for example, the lack of a clear career path for doctoral students and the continuing difficulty of integrating research tasks into clinical practice. Remarkably, these issues are rarely discussed in other healthcare disciplines—such as medicine—where clinical career progression appears to be better established. This suggests that the challenges facing nursing are not unique to academia but are also deeply rooted in the way healthcare systems envision and organise career development. This requires thinking more broadly beyond academic institutions to question how the role of nursing is conceptualised and supported in clinical organisations.

Second, several suggestions point to the need for a broader modernisation of doctoral education. Nursing is expected to open up to other forms of knowledge, methods and cultures of scholarship, even as it remains a core part of the discipline. This transdisciplinary and collaborative vision reflects a desire to break through disciplinary silos and engage meaningfully with other perspectives. The experts argued that doctoral nursing education should develop synergies with other disciplines and across national boundaries to increase both academic relevance and societal impact. Rather than isolating itself, doctoral nursing research should be capable of dialogue—methodologically, conceptually and internationally.

Third, the proposed directions span a continuum between reform and innovation. Some changes require systemic reforms—understood as structural changes to the educational framework, including policy, curricula and institutional governance. These are usually initiated at the decision‐making level and require coordination across the education system. However, other proposals refer to innovation in a narrower sense, i.e., new approaches, methods or technologies introduced to improve or even replace certain elements of doctoral education. Following Adams (1978), this distinction emphasises how reforms operate at a systemic level, while innovations are often bottom‐up initiatives that target selected components of the educational process.

Taken together, these perspectives suggest that the time may be ripe for a structured, Europe‐wide dialogue among experts to usher in a new era of doctoral nursing education—one that is capable of responding to these tensions and evolving needs. Returning to the Bologna Process as a strategic anchor could serve as a catalyst to align doctoral nursing education with broader European objectives of integration and harmonisation. By strengthening a common academic identity and promoting professional development through mobility and mutual recognition, the Bologna framework can provide the much‐needed impetus for revitalising and repositioning doctoral nursing education in Europe—a field that is structurally fragile and underfunded in its current form.

### 4.3 Limitations

This study has several limitations. Although a systematic approach was followed in accordance with the study design, some selection and information bias may have occurred. The literature search was conducted in a limited number of databases, excluding grey and non‐indexed literature, which may have resulted in relevant studies being omitted. In addition, the study was designed at an international level to reflect a Europe‐wide perspective and avoid bias from the perspective of individual countries. Several team meetings were held throughout the process, which helped to minimise the risk of interpretative bias during the data analysis. Nevertheless, such influences may have occurred. The research teams included both experts, i.e., those who had conducted the previous systematic reference reviews (Dobrowolska, Chruściel, Pilewska‐Kozak, et al. 2021; Dobrowolska, Chruściel, Markiewicz, and Palese 2021), and new researchers, all of whom had a background in nursing. While this composition may have limited the inclusion of broader interdisciplinary perspectives, it maintained specificity and relevance to the nursing profession. In addition, the purposive sampling strategy (Patton 2014)—which was initially based on personal networks and later expanded to include experts identified through relevant published articles when the original contacts did not respond—may have influenced the representativeness of the sample. Although the insights gained reflect the opinions of the experts, they may not fully represent the national perspective of the country in question.

## 5 Conclusions

This study offers the most comprehensive and timely synthesis to date of the current state of doctoral nursing education across Europe, integrating findings from a rapid review of the literature with insights from academic leaders actively engaged in the field. The results reveal a dynamic yet fragmented landscape, marked by structural inconsistencies, supervision gaps, insufficient integration of doctoral‐prepared nurses into academic and clinical environments, and a divergence between scholarly discourse and lived professional experiences. While topics such as mentorship, leadership and professional development have gained increasing academic attention, foundational aspects—particularly the candidate–supervisor relationship and the marginalisation of nursing research—remain underexplored. The identification of seven key challenges and six priority areas for improvement underscores the need for coordinated systemic reform. These reforms must include the modernisation of curricula, formalisation of supervision, creation of structured career pathways and enhanced financial and institutional support.

Moreover, expert perspectives highlighted the critical importance of international and interdisciplinary collaboration, policy harmonisation within the Bologna Process and stronger alignment between academic structures and healthcare needs. Addressing both the structural and interpersonal dimensions of doctoral education is essential to cultivating a new generation of nurse researchers equipped to lead in an increasingly complex healthcare and academic environment. Ultimately, this study reinforces the value of bridging empirical evidence with experiential knowledge to inform meaningful, future‐oriented reforms. Ensuring that doctoral nursing education in Europe evolves to meet both academic standards and societal demands will require urgent, deliberate and collaborative action at all levels of the education and policy ecosystem.

### 5.1 Implications

The findings of this study underscore the need for greater coherence and strategic alignment in doctoral nursing education across Europe. Institutions and policymakers should prioritise the development of unified supervision standards, structured career pathways and sustainable funding mechanisms to ensure equitable access and program quality. Without such measures, existing disparities may hinder the development of future nursing scientists and leaders.

Additionally, integrating the lived experiences of academic leaders into educational planning can offer valuable guidance for targeted reforms. The study also highlights the importance of evaluating program outcomes and strengthening collaboration among European countries within the Bologna Process framework to enhance mobility, academic identity and research capacity in the nursing discipline.

## Author Contributions

Conceptualisation: A.P., G.T.E. and B.D. Data curation: A.P., G.T.E., K.B., G.D., G.M., C.M., S.C., A.P.‐K. and B.D. Formal analysis: A.P., G.T.E., K.B., G.D., G.M., C.M., S.C., A.P.‐K. and B.D. Investigation: A.P., G.T.E., K.B., G.D., G.M. and B.D. Methodology: A.P., G.T.E., K.B., G.D., G.M., C.M., S.C. and B.D. Project administration: A.P., G.T.S., A.P.‐K. and B.D. Supervision: A.P., G.T.S. and B.D. Visualisation: A.P., G.T.E, K.B., G.D., G.M. and B.D. Writing – original draft: A.P., G.T.E., K.B., G.D., G.M., C.M., S.C., A.P.‐K. and B.D. Writing – review and editing: A.P., G.T.E., K.B., G.D., G.M., C.M., S.C., A.P.‐K. and B.D.

## Conflicts of Interest

The authors declare no conflicts of interest.

## Supporting information


**Table S1:** Preferred reporting items for systematic reviews and Meta‐analysis guidelines.
**Table S2:** Characteristics of the included studies.

## Data Availability

Data are available upon request.
