# Supplementary material for: Nursing Doctorate Issues, Challenges and Expected Changes Across Europe: A Rapid Review and Experts' Opinion
Source: J Adv Nurs. 2025 Oct 28;82(5):4408–24. doi: 10.1111/jan.70300 (PMC13069255; doi:10.1111/jan.70300)
Supplement: Supplementary file 1 — Table S1: Preferred reporting items for systematic reviews and Meta‐analysis guidelines. Table S2: Characteristics of the included studies. [file JAN-82-4408-s001.docx]

**Supplementary Table 1** Preferred Reporting Items for Systematic Reviews and Meta-Analysis guidelines

| Section/topic | # | Checklist item | Reported on section # |
| --- | --- | --- | --- |
| **TITLE** | | | |
| Title | 1 | Identify the report as a systematic review, meta-analysis, or both. | Title |
| **ABSTRACT** | | | |
| Structured summary | 2 | Provide a structured summary including, as applicable: background; objectives; data sources; study eligibility criteria, participants, and interventions; study appraisal and synthesis methods; results; limitations; conclusions and implications of key findings; systematic review registration number. | Abstract |
| **INTRODUCTION** | | | |
| Rationale | 3 | Describe the rationale for the review in the context of what is already known. | Introduction |
| Objectives | 4 | Provide an explicit statement of questions being addressed with reference to participants, interventions, comparisons, outcomes, and study design (PICOS). | Introduction  *Aim* |
| **METHODS** | | | |
| Protocol and registration | 5 | Indicate if a review protocol exists, if and where it can be accessed (e.g., Web address), and, if available, provide registration information including registration number. | NA |
| Eligibility criteria | 6 | Specify study characteristics (e.g., PICOS, length of follow-up) and report characteristics (e.g., years considered, language, publication status) used as criteria for eligibility, giving rationale. | Methods  *Rapid review* |
| Information sources | 7 | Describe all information sources (e.g., databases with dates of coverage, contact with study authors to identify additional studies) in the search and date last searched. | Methods  *Rapid review* |
| Search | 8 | Present full electronic search strategy for at least one database, including any limits used, such that it could be repeated. | NA |
| Study selection | 9 | State the process for selecting studies (i.e., screening, eligibility, included in systematic review, and, if applicable, included in the meta-analysis). | Methods  *Rapid review* |
| Data collection process | 10 | Describe method of data extraction from reports (e.g., piloted forms, independently, in duplicate) and any processes for obtaining and confirming data from investigators. | Methods  *Rapid review* |
| Data items | 11 | List and define all variables for which data were sought (e.g., PICOS, funding sources) and any assumptions and simplifications made. | NA |
| Risk of bias in individual studies | 12 | Describe methods used for assessing risk of bias of individual studies (including specification of whether this was done at the study or outcome level), and how this information is to be used in any data synthesis. | NA |
| Summary measures | 13 | State the principal summary measures (e.g., risk ratio, difference in means). | NA |
| Synthesis of results | 14 | Describe the methods of handling data and combining results of studies, if done, including measures of consistency (e.g., I^2^) for each meta-analysis. | NA |
| Risk of bias across studies | 15 | Specify any assessment of risk of bias that may affect the cumulative evidence (e.g., publication bias, selective reporting within studies). | NA |
| Additional analyses | 16 | Describe methods of additional analyses (e.g., sensitivity or subgroup analyses, meta-regression), if done, indicating which were pre-specified. | NA |
| RESULTS | | | |
| Study selection | 17 | Give numbers of studies screened, assessed for eligibility, and included in the review, with reasons for exclusions at each stage, ideally with a flow diagram. | Methods  *Rapid review* |
| Study characteristics | 18 | For each study, present characteristics for which data were extracted (e.g., study size, PICOS, follow-up period) and provide the citations. | Results  *Rapid review* |
| Risk of bias within studies | 19 | Present data on risk of bias of each study and, if available, any outcome-level assessment (see Item 12). | NA |
| Results of individual studies | 20 | For all outcomes considered (benefits or harms), present, for each study: (a) simple summary data for each intervention group and (b) effect estimates and confidence intervals, ideally with a forest plot. | Results  *Rapid review* |
| Synthesis of results | 21 | Present results of each meta-analysis done, including confidence intervals and measures of consistency. | NA |
| Risk of bias across studies | 22 | Present results of any assessment of risk of bias across studies (see Item 15). | NA |
| Additional analysis | 23 | Give results of additional analyses, if done (e.g., sensitivity or subgroup analyses, meta-regression [see Item 16]). | NA |
| DISCUSSION | | | |
| Summary of evidence | 24 | Summarize the main findings including the strength of evidence for each main outcome; consider their relevance to key groups (e.g., health care providers, users, and policy makers). | Discussion |
| Limitations | 25 | Discuss limitations at study and outcome level (e.g., risk of bias), and at review level (e.g., incomplete retrieval of identified research, reporting bias). | Discussion |
| Conclusions | 26 | Provide a general interpretation of the results in the context of other evidence, and implications for future research. | Conclusions |
| FUNDING | | | |
| Funding | 27 | Describe sources of funding for the systematic review and other support (e.g., supply of data); role of funders for the systematic review. | Funding statement |

**Legend**

NA, Not Available

**Supplementary Table 2** Characteristics of the included studies

| **No** | **Author(s)/**  **year/**  **country** | **Aim(s) of the study** | **Study design** | **Sample (n)** | **Main findings** | **Recommendations** |
| --- | --- | --- | --- | --- | --- | --- |
| 1 | Cleary et al.  2023  Australia | To synthesize the existing evidence on the experience of mentoring doctoral students (PhD, DNP, DNS, EdD) in nursing students to capture the experiences of doctoral nursing students and academic mentors with mentoring; to identify positive and negative attributes of mentors and the mentor-student relationship, and to assess the benefits and barriers of mentoring. | Scoping review | 30 articles  Europe:  Ireland (n=1)  Other countries:  USA (n=27)  Japan (n=2) | The expectations of students entering a mentorship program, the benefits and barriers experienced by mentors and students, the mentor-student relationship and the characteristics of interventions used to mentor PhD, DNP, DNS and EdD students. | Developing structured mentoring program  Using of guidelines, norms, and standards of mentoring doctoral students |
| 2 | Dalfo-Pibernat et al.  2020  Spain | To determine the degree of knowledge of primary care (PC) nurses on the principles of self-management of heart failure (HF) and variables associated with this. | Observational, cross-sectional  descriptive study | 216 PC nurses – 16 with PhD degree | Having a PhD degree (OR: 36.4, 95% CI: 2.8–468.2, p = 0.006) and having received previous HF training (OR: 19.8, 95% CI: 1.4–279.3, p = 0.026) were significantly associated with an adequate knowledge of HF education principles. | NR |
| 3 | Hafsteinsdóttir et al.  2025  The Netherlands | To evaluate changes in leadership practices, career development and research productivity of PhD-prepared nurses after participating in the Leadership Mentoring in Nursing Research (LMNR) 2.0 program | Pretest–posttest online survey | 17 PhD-prepared nurses applied for the LMNR 2.0 program | The fellows:  (1) demonstrated improved leadership development in all leadership practices inventory;  (2) advanced into higher positions;  (3)increased their research productivity. | Exploring how to better support PhD-prepared nurses in achieving successful academic careers  PhD-prepared nurses are crucial to advancing nursing and healthcare by generating and implementing new knowledge into clinical practice and educating nurses in the future  National and international leadership and mentoring programs are essential to help PhD-prepared nurses become successful scientists and the future leaders in nursing |
| 4 | Henshaw et al.  2025  UK | To explore the routes to doctoral level qualifications in the nursing profession. | Discussion paper | NR | (1) There is an increasing need and desire for nurses to pursue doctoral-level education, yet the number of nurses with such qualifications remains low.  (2) Nursing professionals must collaborate with universities, workforce development bodies and employers to support nurses at all levels in considering doctoral study.  (3) In the UK, the NIHR’s initiatives to expand research opportunities for nurses through fellowships and career development programs are encouraging and beneficial to both professional development and patient care. However, the reluctance of the NIHR to fully recognize professional doctorates, despite their equivalence and relevance to participation in certain programs, remains an issue that requires attention.  All doctoral programs offer prestigious and rigorous academic qualifications. Despite differences in structure and delivery, they are subject to rigorous quality assurance standards and should be valued equally in academic research and healthcare. | Improving in organizations, such as universities, workforce development and employers to consider how prepare and support nurses for study at doctoral level |
| 5 | Holmberg 2024  Sweden | To analyze nursing research articles indexed as Swedish in the Web of Science Core Collection from the database’s inception to the time of data collection | Bibliometric analytical and mapping approach | 7213 articles indexed as Swedish | The findings reveal high productivity among Swedish nursing researchers, likely due to international (32%) and university collaborations. Norway, the USA, and Australia had the most collaborations. Only 299 (4.1%) articles were authored by a single individual, and the research spanned diverse clinical and nursing-specific areas. The most cited articles focused on methodological or conceptual aspects. Current research trends encompass health literacy and telehealth/digital health. | Improving extensive national and international collaborations as successful factors |
| 6 | Lino et al. 2022  Portugal | To understand the contributions of the Bologna Process to academic-professional mobility of Nursing | Descriptive, exploratory qualitative research | 6 PhD nursing professors on high education | Three perspectives are presented:  (1) mobility and internationalization as prerequisites for strengthening the economic bloc;  (2) mobility and internationalization contribute to the consolidation of a European identity;  Diploma Supplement is a fundamental aspect of academic and professional mobility. | Implementing guidelines for the harmonization of university systems with a view to the premises of increasing Europe’s competitiveness  Promoting internal and external mobility  Leading nursing to a major change in its academic configuration and spectrum of action at the international level |
| 7 | McBride-Henry et al. 2024  New Zealand | To identify graduate outcome domains that can be applied internationally to benchmark and evaluate PDPN. | An exploratory study was carried out in three phases:  (1) scoping review;  (2) document analysis;  (3) thematic analysis. | 12 papers  Europe:  UK (n= 7)  Poland (n= 1)  Other countries:  USA (n=1)  Australia (n=3)  3 European universities: UK; NL; NZ | A scoping review revealed three key themes:  (1) Personal transformation;  (2) Critical self-awareness;  (3) Bridging the theory–practice divide.  A document analysis of three universities' Professional Doctorate Programs (UK, NL, NZ) revealed similarities in graduate outcomes (research competences, ethical behaviors, professional leadership).  The third research phase identified five graduate outcome domains:  (1) Personal achievement;  (2) Critical self-awareness;  (3) Professional identity;  (4) Professional citizenship;  (5) Discipline, research, information literacy, and community-based academic practice. | Needing for a standardized evaluation process and efforts to ensure consistency and quality across programs  Strengthening the transnational networks for professional doctorates in nursing sector to facilitate ongoing collaboration.  Enhancing the recognition and value of PDPs for the discipline of nursing  Addressing the challenges of evaluating and standardizing PDPNs globally with a cooperative approach |
| 8 | Met et al. 2022  France | To understand the place and experience of ND/NDS in healthcare organizations.  To understand, through biographical journeys, the elements that trigger doctoral processes at the work organization level.  To identify organizational resources that allow NDS to conduct their research and integrate it with their other activities.  To characterize the working relationships ND/NDS have with peers, colleagues and management. | Two-stage mixed methodology.  Questionnaires and  semi structured  interviews, interviews  and in situ observations | 79 participants  45semi structured  interviews with nurses;  10 interviews with health managers and chief nurses;  27 hours of in situ observations with research coordinators | Work organization in some departments (intensive care units, oncology, and psychiatry) promote the development of scientific expertise among nurses.  Elements that promoted the professional and academic development of ND/NDS included the potential for medical teaching in healthcare services, participation in research projects, and proximity to medical professionals.  Poor visibility and recognition of nursing doctoral courses in French health organizations resulted from a lack of task missions, suitable posts, and poor integration into the nursing profession and research | Implementing in the healthcare organizations the learning process  Organizing care to allow nurses participate in research  Legislating for nurse ratios to facilitate nurse availability for cross-sectional activities such as research  Integrating research activity into professional nursing practice, education, and management,  Valuing work practices through the implementation of care protocols derived from ND/NDS theses  Improving integration of research activities by the nursing profession and promoting nurse scientific careers |
| 9 | Negarandeh and Khoshkesht,  2022  Iran | To investigate the expected roles of the Doctor of Philosophy graduated nurses. | Scoping review | 30 Articles and 2 Books  Europe:  UK (n=3)  Other countries:  Australia (n=1)  Canada (n=2)  China (n=1)  Iran (n=9)  USA (n=14) | The educational goals for training Ph.D. graduates differ due to discrepancies in defined roles and responsibilities.  (1) Achieving an academic career or other employment in healthcare services, ward management, policy development, or leadership.  (2) Promoting health in communities at higher executive and managerial levels without a direct clinical role;  (3) Preparing nursing students to produce new knowledge, develop the profession, and improve the quality of healthcare and health policies based on research studies. | Thinking about the consistency between curriculums and roles  Establishing a better relationship between academic settings, educational programs and organizations providing job opportunities |
| 10 | Orton et al. 2022  Sweden | To explore published articles concerning the clinical contributions of RN/PhDs and their impact on the quality and improvement of nursing care. | Systematic review | 12 studies  Europe  Denmark (n=2)  Nordic Countries (n=1)  Sweden (n=1)  The Netherlands (n= 1)  Other countries: Australia (n=2)  USA (n=5) | Three categories emerged from the analysis:  (1) Bridge between theory and practice: clinical resource for nurses and other professionals (interpreting and discussing scientific articles related to complex patient care), as well as for patients and their families;  (2) Leadership role in developing nursing practices that improve quality of care;  (3) Professional Tradition: The role of RN/PhDs in clinical care has yet to be clearly defined. Limited career opportunities are one important reason for not combining clinical and academic work. | Healthcare managers should consider how to provide RN/PhDs with adequate opportunities to combine research and clinical practice, with the aim of improving nursing practices  Clarifying the defined clinical positions in which RN/PhDs can take full advantage of their expertise |
| 11 | Ottonello et al.  2024  Italy | To report a reflection on the role, challenges and opportunities for nurses with advanced education in research outside the academic field. | Discursive paper | Two PhD-prepared nurses (70% and 30% of their monthly work time spent on patient care and research) | This "hybrid" figure links theory, clinical practice, and the organizational components of quality improvement management, training, and research.  This solution represents one way in which nurses with advanced training in the research field can be employed and valued, not only in academia, but also in healthcare organizations in managerial and clinical roles. | Investing in the continuing education of nurses, with an emphasis  on a long-term perspective, can promote a research culture as a  tool for continuous improvement in care  Adopting this long-term perspective, and moving beyond the practice of immediate interventions, could facilitate the establishment of enduring structures and partnerships could be created |
| 12 | Sanders et al.  2022  UK | To explore, establish and implement a model for supporting post-doctoral clinical academic career opportunities for NAHP and HCS at St Bartholomew's Hospital. | Discussion paper | A multidisciplinary group devised the model which was approved by the St Bartholomew’s Hospital Executive Board. | A model was developed that includes the following: a plan for each individual; planning the postdoctoral role at the time of PhD planning; providing one day a week of protected research time for at least 12 months after PhD completion; an appropriate honorary academic contract at a partner university; and integrated clinical and academic supervision throughout. Other key components include senior clinical and executive board support, as well as an existing, vibrant research culture within our organization. | Providing dedicated time and mentorship within a supportive and  established research environment  Creating a sustainable infrastructure for increasing post-doctoral NAHP and HCS workforce that can provide the clinical and academic expertise to implement evidence-based practice and generate new knowledge |
| 13 | Skela-Savič et al.  2020  Slovenia | To describe the presence, characteristics and content of EBP course in nursing study programs (bachelor's, master's, and PhD cycles) in six European countries | Mixed methods including descriptive cross-sectional and descriptive qualitative research methods | 162 faculties:  Czech Republic (n=15)  Greece (n=7)  Italy (n=44)  Poland (n=12)  Slovenia(n=5) Spain (n=79) | Fifteen doctoral study programs are offered by faculties in four countries (the Czech Republic, Italy, Poland, and Slovenia). Six of them (37.5%) are three-year programs worth 180 ECTS, and nine (56.3%) are four-year programs worth 240 ECTS.  Only six faculties (37.5%) in the Czech Republic and Slovenia include subjects or modules on "Evidence-Based Practice (EBP) in Nursing or Health Care" in their doctoral programs, which include an obligatory EBP subject. On average, seven steps of EBP in nursing are covered in 7.43 hours. | Developing guidelines on the standardization of EBPs’ teaching approaches and content in all three cycles of higher education  Implementing of EBP teaching at the master's and doctoral levels of nursing curricula |
| 14 | Sterkenburg et al.  2025  The Netherlands | To reach consensus on professional competencies, develop an instrument to measure professional competencies of PhD prepared nurses and assess the content validity of the instrument, using a two-phased Delphi and validation method. | A Delphi study and content validity study | 28 PhD Nurses | This study developed a 13-item self-assessment instrument, the Postdoctoral Nurses Competence Scale (PNCS), to measure the professional competencies of PhD-prepared nurse researchers. The instrument showed excellent content validity. | Raising awareness about the key competencies required after completing a PhD program and guiding PhD students to focus on developing the skills needed for a successful doctoral research career  Measuring outcomes of mentoring programs designed for PhD prepared nurse researchers  Providing a structured approach to competency development and career progression |
| 15 | Stolldorf et al.  2022  USA | To report on methods and metrics previously used in assessing postgraduate outcomes across disciplines and provide recommendations regarding methods and metrics for conducting postgraduate evaluation of research-intensive nursing science PhD programs. | Literature review | 9 articles  Europe:  Sweden (n=1)  Other countries: USA (n=8), one cross-sectional survey from 20 countries | Only a few reports deal with the evaluation of doctoral programs, especially those that describe in detail the type and number of questions and the general survey methods of the questionnaire. These reports focus on postgraduate evaluations of Ph.D. nursing programs, M.D./Ph.D. programs and other discipline-specific programs. Evaluation methods used in PhD programs in nursing include surveys on employment patterns, curriculum vitae, career development, scholarly productivity, professional leadership, number of presentations and publications, peer review activities, grants funded and/or submitted, and questions aimed at understanding perceived attainment of knowledge. | Establishing postgraduate PhD program evaluation as part of the overall evaluation plan  Developing standardized core program evaluation metrics focused on three key domains: scholarship and funding, employment patterns and leadership roles, and program characteristics  Continuing AACN national task force efforts  Establishing a national shared evaluation data repository |
| 16 | Taneva et al.  2023  Bulgaria | To investigate and compare the similarities and differences in the training programs of nurses in selected European Higher Education Area member (EHEA) member countries | Comparative analysis on research articles, internet databases, and emails sent to nursing education institutions. | 13 articles  23 emails  56Internet databases  19 EHEA member countries | EHEA countries considered: Bulgaria, Germany, Greece, Denmark, Estonia, Ireland, Sapin, Italy, Cyprus, Latvia, Portugal, Russia, North Macedonia, Slovenia, Finland, France, Czech Republic, Switzerland, Sweden.  Estonia, Latvia, and France did not have PhD programs in nursing, while the other EHEA countries considered in the analysis did. | Unifying nursing education within the European Higher Education Area enables nurses to practice their profession across the European Union |
| 17 | Tikkanen et al.  2025  Finland | To gain a better understanding of the level and the differences in doctoral supervisors’ occupational wellbeing;  To understand whether the doctoral supervisors’ occupational wellbeing is related to the support they received from the scholarly community, or their supervisory experience in terms of supervisory competencies, supervisory interaction | Survey | 561 doctoral supervisors from a research-intensive, international, multidisciplinary university in Finland.  27% Health Science  16% Environmental, food and biological sciences  38% Humanities and Social Sciences  18% Natural sciences | Differences in doctoral supervisors’ work engagement, and burnout symptoms:  Supervisors in various positions at the university reported different levels of work engagement and burnout symptoms. Health science doctoral supervisors reported high levels of work engagement, moderate levels of stress, burnout, and exhaustion, and low levels of cynicism. Women reported higher levels of exhaustion than men. No differences based on supervisees' study status were found in supervisors' work engagement, exhaustion, or cynicism. Supervisors who reported that their supervisees were engaged in a research group experienced higher levels of work engagement and lower levels of cynicism. No differences based on supervisees' research group status were detected in supervisors' exhaustion. Supervisors who perceived the number of supervisees as suitable reported higher levels of work engagement and lower levels of cynicism and exhaustion.  The associations between supervisors’ occupational well-being and support from the scholarly community and supervisory experience:  (1) The supervisors’ perceptions of the research community were directly related to their confidence in their supervisory competencies.  (2) The supervisory competencies were related to better quality supervisory interactions, increased work engagement, and decreased burnout symptoms.  (3) The supervisors’ perceptions of the quality of supervisory interactions did not contribute to work engagement or burnout. | Investing in the development of professional support practices within the research community, and supervisors’ competencies |
| 18 | Tyndall et al.  2021  USA | To examine the empirical literature on threshold concepts identified in doctoral education across multiple disciplines.  To explore factors affecting doctoral students’ conceptual threshold crossing.  To recommend programmatic strategies for doctoral nursing programs to build capacity for conceptual threshold crossing in PhD students completing their doctorate. | Integrative Review | 20 qualitative studies  Europa:  UK (n=6)  Other countries: Australia (n= 6)  New Zealand (n=5)  United States (n=2) | Threshold concepts in doctoral education emerged during the development stage, which includes conducting a literature review, linking theoretical perspectives through the literature review, applying theory for research purposes, generalizing from specific research to a theory, framework, or conceptual model, formulating a convincing argument, selecting appropriate research topics with objectives, writing research questions, conceptualizing appropriate control conditions, and designing methods. These concepts also emerged during the dissemination stage, which includes demonstrating significance. Understanding the limitations of their research, creating knowledge from their findings, and situating their research contributions within the current literature. Relating to doctoral writing ("talking to think," developing self-efficacy, and writing up qualitative data) and during the research implementation stage (analyzing research data). One study provided a comprehensive view of doctorateness. Other researchers presented the concept of doctorateness as troubling, particularly for international students.  Factors affecting conceptual threshold crossing include writing development, the presence of a community, and faculty influence. | Investigating what approaches are most effective at supporting the development of research and writing skills in PhD nursing programs  Implementing group discussions  Using of writing to connect thinking and writing, structured reading, and use ‘talking to think’ to clarify student thinking |
| 19 | Van Dongen et al.  2022  The Netherlands | To explore the leadership experiences and the influence of leadership on the career development of PhD-prepared nurses working in hospitals | Qualitative descriptive study with semi-structured interviews and  thematic analysis | 12 PhD nurses employed at a clinical hospital department from seven Netherlands hospitals | Three themes addressing leadership experiences were identified:  (1) Leadership is needed for career development: it describes how participants took the initiative and received support from colleagues and mentors;  (2) Practicing leadership behaviors: it describes leadership characteristics and feelings associated with leadership;  (3) Leadership Influenced by the hospital setting: it describes the challenges of nursing research cultures and infrastructures, the need for suitable positions, managerial support, and collaborations. | Developing more international leadership and mentoring programs |
| 20 | Van Dongen et al.  2024  Finland | To integrate the research literature on careers, career development and factors influencing career development of nurses with doctoral degrees | Integrative review | 22 studies  European:  The Netherlands (n= 2)  Sweden (n=1)  UK (n=1)  Other countries:  Jordan (n=2)  USA (n= 16) | Three categories and nine themes emerged:   1. Career: doctorally prepared nurses need to prioritize work within different positions; 2. Career development: the need to determine career goals after the doctorate and further develop competencies;   Factors influencing career development: intrinsic motivation to improve health care and nursing education, available support sources, professional development programs, work–life balance, organizational infrastructures for career advancement and competition and hostile treatment among colleagues. | Developing leadership competences and opportunities as part of doctoral education program to establish advanced and sustainable careers  Focusing on what competences are needed to build suitable careers  Facilitating the leadership development of PhD-prepared nurses by hospital, providing access to educational programs for leadership development, mentoring as well as developing structures for collegial support and research collaboration  Creating organizational structures and policies to support career development  Implementing advanced career frameworks by hospital with integrated joint positions in research, quality improvement, administration and access to educational programs for PhD-prepared nurses as well as policies facilitating research activities |
| 21 | Van Dongen et al.  2024  Finland | To evaluate changes in leadership practices, professional and research competencies, and career development of PhD-prepared nurses and doctoral students after participating in the Nurse-Lead program. | Pre and post-test program evaluation with a convergent mixed methods design | 30 of PhD-prepared nurses and doctoral students from Finland, Germany, Iceland, Lithuania, the Netherlands, and Portugal | The participants showed significant improvements in the following areas:  (1) all leadership practices, by gaining knowledge of leadership theories and practices, and focusing on transferring that knowledge to work practices;  (2) professional competencies, improving team management and networking skills;  (3) Research competencies, such as improving scientific thinking, research management, and new strategies for branding their research. They also improved their knowledge of research ethics and English language skills.  (4) Career development by supporting the participants to develop a vision, plans for their careers. It also explored career decisions to reach career goals and to gain insights toward future ambitions. Eight participants moved into new positions during the program. | Conducting robust and longitudinal international research to gain insight into the careers and career outcomes of both PhD and DNP-prepared nurses  Gaining insight into the competencies needed to build successful careers in various settings  Exploring preferred strategies to support the professional and career development from the perspective of PhD-prepared nurses |
| 22 | Wong et al. 2025  UK | To identify a set of evidence-based key considerations, with nursing and midwifery PhD students, for education facilities and educators when developing support for them in addressing their academic writing needs and increasing their confidence in writing | Scoping review | 11 papers  European:  UK (n=1)  Other countries:  Australia (n=6)  Canada (n=1)  USA (n=3) | Six themes identified a key consideration for developing effective academic writing support:   1. Modes of delivery; 2. Barriers to writing; 3. Accountability and productivity; 4. Building group identity, collegiality and a sense of community; 5. Peer review;   (6) Behavioral change in writing practice. | Exploring how to best address the strategies to support academic writing  Facilitating interprofessional learning and collaboration  Providing doctoral students with the opportunity to actively engage in conversations with their peers, share writing commitments, and develop a sense of achievement to build confidence to maintain regular and productive academic writing  Focusing on the nursing and midwifery students’ educational needs for academic writing support |
| 23 | Zerbe S. 2025  Italy/Germany | To critically reflect on different PhD qualification formats (individual PhD, structured PhD program and Junior research group) focusing on their strengths and weaknesses, particularly in Germany and Italy | Discussion paper | NR | The three doctoral pathways—the Individual PhD, the Structured PhD Program, and the Junior Research Group—differ in terms of structure, responsibilities, and the level of support provided to doctoral candidates.  Admission and recruitment: for the Individual PhD model, students are recruited directly by a supervisor. For the Structured PhD Program, admission is managed by a selection committee. For Junior Research Groups, admission is managed by the research group leaders.  Duration and program structure: individual PhD: No formal time limit. Structured programs: Typically, last 3–6 years. Junior Research Groups: 3–5 years, depending on available funding. The structured programs and junior groups are more formalized, with features such as credits, regular progress reviews, and structured milestones, which are often lacking in the individual model.  Research focus and funding: individual model: the research topic may be proposed by the student or shaped by a pre-existing funded project. Structured and Junior Group Models: the topic is usually predefined in the call for applications or linked to an ongoing project. Funding is more consistently available through fellowships or research grants in the structured and junior group formats, whereas funding is not guaranteed in the individual format.  Supervision and Support: in the individual model, supervision is typically carried out by one academic or a small team. A high degree of personal responsibility falls on the student. Structured programs and junior groups involve at least two supervisors and often a coordinating figure, offering more systematic guidance and integration into research environments.  Internationalization, interdisciplinarity, and skill development: internationalization and interdisciplinarity are more prominent in structured programs and junior groups, which promote collaborative networks and multidimensional training. These models explicitly support the development of hard and soft skills, whereas in the individual model, this largely depends on the student’s initiative.  Personal responsibility and risk of isolation: the individual PhD program requires high levels of autonomy and motivation but may lead to isolation, slow progress, and psychological strain, as reflected in research on burnout. Structured programs and collaborative groups provide more support, reducing dropout risks by fostering peer interaction and more guided progression. Although the individual PhD offers more flexibility and independence, structured programs and junior research groups provide a more organized, supportive, and collaborative academic environment that is better suited to developing comprehensive competencies and ensuring successful completion of the PhD program. | Diversifying approach based on discipline and context  Assessing qualitatively and quantitatively the three pathways to attain a PhD through the perspectives of doctoral candidates, supervisors, and administrative staff. |

**Legend:** AACN, American Association of Colleges of Nursing; DNP, Doctor Nursing Practice; DNS, Doctor Nursing Science; EBP, Evidence Based Practice; ECTS, European Credit Transfer System; EdD, Doctoral Education in Nursing; EHEA, European Higher Education Area member; HCS, Healthcare Scientists; HF, Heart Failure; LMNR, Leadership Mentoring in Nursing Research; NAHP, Nurses Allied Health Professionals; ND, Nurse Doctor; NDS, Nurse Doctoral Students; NIHR, National Institute for Health and Care Research; NL, Netherlands; NR, Not Reported; NZ, New Zealand; PC, Primary Care; PDPN, Professional Doctorate Programs for nurses; PhD, Doctor of Philosophy in Nursing; RN, Registered Nurse; UK, United Kingdom; USA, United States of America
